# Mucopolysaccharidosis type VI (Maroteaux-Lamy syndrome): defining and measuring functional impacts in pediatric patients

**DOI:** 10.1186/s13023-021-02113-8

**Published:** 2021-12-02

**Authors:** Beth Leiro, Dawn Phillips, Melanie Duiker, Paul Harmatz, Sharon Charles

**Affiliations:** 1 Phillips Consulting, Chapel Hill, NC USA; 2 Paradigm Biopharmaceuticals Limited, Melbourne, VIC Australia; 3 UCSF Benioff Children’s Hospital, Oakland, CA USA

**Keywords:** Clinical Outcome Assessment, Focus Groups, Mucopolysaccharidosis VI, Pain, Patient Reported Outcome Measures, Pediatrics

## Abstract

**Background:**

Research about pediatric patients’ perspective on mucopolysaccharidosis type VI (MPS VI) and its impact on daily life is limited. We aimed to identify the disease concepts of interest that most impact function and day-to-day life of pediatric patients with MPS VI, and to consider clinical outcome assessments (COAs) that may potentially measure meaningful improvements in these concepts.

**Methods:**

Potential focus group participants were identified by the National MPS Society (USA) and invited to participate if they self-reported a clinician-provided diagnosis of MPS VI and were 4 to 18 years, receiving enzyme replacement therapy (ERT), and available to attend a 1-day focus group with their caregiver in Dallas, TX, USA. The focus group consisted of a series of polling and open-ended concept elicitation questions and a cognitive debriefing session. The discussion was audio recorded, transcribed verbatim, and analyzed to identify disease concepts of interest and functional impacts most relevant to participants.

**Results:**

Overall, caregivers (n = 9) and patients with MPS VI (n = 9) endorsed that although their children/they receive ERT, residual symptoms exist and impact health-related quality of life. The key disease concepts of interest identified were impaired mobility, upper extremity and fine motor deficits, pain, and fatigue. Pain was unanimously reported by all patients across many areas of the body and impacted daily activity. Key disease concepts were mapped to a selection of pediatric COAs including generic measures such as PROMIS®, PODCI, CHAQ, and PedsQL™. Caregivers endorsed the relevance of PODCI and PROMIS Upper Extremity, Mobility, and Pain items and all patients completed the NIH Toolbox Pegboard Dexterity Test. Additional COAs that aligned with the disease concepts included range of motion, the 2- and 6-min walk tests, timed stair climbs, Bruininks-Oseretsky Test of Motor Proficiency, 2nd edition, grip strength, pain visual analog scale, and the Faces Pain Scale-Revised.

**Conclusion:**

An MPS VI focus group of pediatric patients and their caregivers identified impaired mobility, upper extremity and fine motor deficits, pain, and fatigue as key disease concepts of interest. These disease concepts were mapped to existing pediatric COAs, which were provided to the group for endorsement of their relevance.

## Background

Mucopolysaccharidosis type VI (MPS VI; Maroteaux-Lamy syndrome) is a rare, lysosomal storage disorder in which inherited mutations lead to deficient N-acetylgalactosamine 4-sulfatase activity and the progressive accumulation of partially degraded glycosaminoglycans in various organs and tissues [1]. The resulting cellular injury from glycosaminoglycan accumulation is associated with diverse, often debilitating, clinical manifestations that can progress rapidly in some patients [2].

The musculoskeletal, cardiorespiratory, ocular, auditory, and sometimes neurologic manifestations of MPS VI can profoundly impact mobility, activities of daily living (ADL), and health-related quality of life (HRQoL) [3–5]. Treatment of MPS VI with enzyme replacement therapy (ERT) and multidisciplinary management of disease manifestations (eg, adaptive or supportive devices, physiotherapy, surgery) slows progression of and/or provides relief from some disease manifestations [5]. However, the need for novel treatments for MPS VI continues, and, increasingly, the patient’s perspective on their disease and how it impacts their daily life is being used to strengthen clinical research and evaluate “meaningful” effects [4].

Research about pediatric patients’ perspective on MPS VI and its impact on daily life is limited [6–9]. MPS VI is a rare disease and is frequently studied with other MPS types. Although the MPS types share many clinical features [4], differences among types warrant delineation. Similarly, disease presentation and rates of progression vary widely, and extrapolating findings from adult to pediatric patients is not possible.

Engaging pediatric patients with MPS VI and their families provides insight into their perspectives and allows methodical characterization of their disease symptoms and impacts on daily life. The objectives of this project were to identify the disease concepts of interest that are most impactful on function and day-to-day life of pediatric patients with MPS VI participating in a focus group, and to consider clinical outcome assessments (COAs) that may potentially measure meaningful improvements in these concepts in clinical trials of new treatments for MPS VI.

## Methods

### Literature review

Key pediatric MPS VI disease concepts of interest and COAs currently used in MPS VI clinical studies and practice were identified in a review of MPS VI-specific literature and their bibliographies retrieved from searches of PubMed and Google Scholar (general search terms: MPS VI, pediatric, COA, clinical presentation, ambulation, enzyme replacement therapy; limits: English language and human species). The review also included poster presentations from professional conferences and content on MPS patient advocacy websites. MPS VI clinical studies listed in clinicaltrials.gov were also reviewed for outcome measures. All searches were done in May 2019.

### Focus group

Potential focus group participants were identified by the National MPS Society (USA; https://mpssociety.org/) and were invited to participate if they self-reported a clinician-provided diagnosis of MPS VI, were 4 to 18 years of age, were currently receiving ERT, and were available to travel and attend a 1-day focus group in person with their caregiver. Patients with MPS VI and/or caregivers provided consent/assent to participate in the focus group.

The focus group was held in-person on 22 June 2019 in Dallas, TX USA. The focus group was conducted by experienced qualitative interviewers (DP and BL) and Sponsor representatives were present (SC and MD). Patients with MPS VI and their caregivers participated in the discussion, which was audio recorded.

The focus group consisted of a series of polling and open-ended concept elicitation questions and a cognitive debriefing session. Polling and open-ended concept elicitation questions focused on key disease concepts of interest, including mobility, arm and hand function, self-care, dressing, endurance, and pain, identified during the literature review. The questions were designed to understand the range of symptoms, the impact of symptoms on ADLs and HRQoL, and how disease concepts varied by age, disease severity, and ambulatory status. Questions were directed either to all participants, caregivers only, or patients only.

For the cognitive debriefing session, patients ≥ 8 years of age and caregivers were asked to complete the Pediatric Outcomes Data Collection Instrument (PODCI) [10] questionnaire and selected Patient Reported Outcome Measurement Information System (PROMIS®) [11] instruments (pain behavior, pain interference, mobility, upper extremity) to validate the relevance and applicability of these instruments overall and their individual items. To elicit discussion after completing these instruments, participants were asked to describe their thought process as they interpreted and responded to each item, and were asked probing questions, such as “How relevant is this question to you?” and “Tell me what this question means to you”, to determine if they endorsed the applicability of the item. In addition, the National Institutes of Health (NIH) Toolbox Pegboard Dexterity Test was administered to assess its relevance and feasibility for pediatric patients with MPS VI.

### Analysis

The focus group discussion was transcribed verbatim. All patient/caregiver information was de-identified before providing any content to the Sponsor. The transcripts were analyzed to identify disease concepts of interest and functional impacts most relevant to participants. Responses to polling questions were determined using polling software supplemented by the meeting transcript and notes collected in real time.

A conceptual model was developed by using data from the literature review, the focus group and refining the HRQoL conceptual model for patients with MPS developed by Hendriksz et al. [4] to define MPS VI measurement considerations and to include age-appropriate functional tasks.

## Results

### Focus group participant characteristics

Nine patients with MPS VI and 9 caregivers participated in the focus group. All patients and caregivers spoke English. Patients were 4 to 18 years of age, were predominantly female (n = 8), were receiving ERT (n = 9), and had not received hematopoietic stem cell transplantation (n = 9). Two patients had slowly progressing MPS VI (age: 14 and 16 years) and 7 had classic MPS VI (age: 4 to 18 years). Caregivers were predominantly female (n = 8).

### Disease concepts of interest and functional impacts identified by the focus group

Key focus group concepts of interest identified were pain, impaired mobility, upper extremity and fine motor deficits, and fatigue. Overall, the most bothersome/challenging aspects of MPS VI nominated by caregivers were spread across mobility (33%) independence in daily activities (33%), sleep (22%) and pain (11%), whereas the second most bothersome/challenging aspect nominated was predominantly pain (67%) (Table 1).

**Table 1:** Aspects of MPS VI function that caregivers find the most bothersome or challenging for their child

Aspect	Most bothersome/challenging			
	First	Second	Third	Fourth
Mobility including ambulation and stairs	3 (33.3%)	2 (22.2%)	1 (14.3%)	1 (14.3%)
Independence in dressing and using the bathroom	3 (33.3%)	0 (0%)	1 (14.3%)	0 (0%)
Fine motor tasks like writing, using a computer mouse/keyboard or grasping small items	0 (0%)	0 (0%)	1 (14.3%)	4 (57.1%)
Pain	1 (11.1%)	6 (66.7%)	0 (0%)	0 (0%)
Sleep	2 (22.2%)	1 (11.1%)	0 (0%)	2 (28.6%)
Fatigue	0 (0)	0 (0)	4 (57.1%)	0 (0%)
Total	9	9	7	7

Not all participants responded to polling questions because of fatigue, care provision, or other reasons

### Pain

**Quote Pain and Inattention:** “Because of pain I'm hardly focused on anything. I can't pay attention.”

**Quote Moving Despite Pain:** “They can do it but you can see that it's difficult and painful for them.”

Pain was identified by one caregiver as the most challenging symptom, and by 6 caregivers as the second most challenging symptom (Table 1). Pain was present in hands, wrists, shoulders, knees, hips, and back. Most caregivers reported that pain impacted their child’s sleep and ability to participate in sports and recreational activities and complete schoolwork (Table 2). Most patients indicated that pain affected their ability to fall and/or stay asleep at night (always, n = 1/8 [12.5%]; sometimes, n = 5/8 (62.5%), [17%]; never, n =2/8 [25%]).

**Table 2:** Caregiver perspectives on the impact of pain on their child

Pain limits my child’s…	Never	Almost never	Some-times	Almost always	Always	Total
Ability to complete schoolwork	3 (37.5 %)	1 (12.5%)	3 (37.5)	1 (12.5%)	0	8
Ability to fall or stay asleep at night	2 (25%)	0	5 (62.5%)	0	1 (12.5%)	8
Participation in sports and recreational activities	2 (22.2%)	0	4 (44.4%)	2 (22.2%)	1 (11.1%)	9

Not all participants responded to polling questions because of fatigue, care provision, or other reasons

In the cognitive debriefing session, caregiver and patient responses on the PROMIS Parent Proxy Short Form v2.0—Pain Interference 8a and PROMIS Pediatric Short Form v2.0—Pain Interference 8a, respectively, underscored the presence and impact of pain in MPS VI. Caregivers endorsed all 8 items, which evaluate the impact of pain on mobility, sleeping, paying attention, and having fun. Additionally, all caregivers endorsed all 8 items of the PROMIS Parent Proxy Short Form v1.0—Pain Behavior 8a, which describes strategies used to manage pain, such as moving more slowly, asking for help, laying down, and requesting medicine.

### Impaired mobility

**Quote Mobility and Social Impact:** “They'll cry, they'll get frustrated that everybody ran from this area of the playground to the other and they couldn't keep up and they're yelling at them to wait for them and it's frustrating for them, but they still do it”.

**Quote Mobility Skills:** “Right now, we are mostly struggling with walking longer distances and they're not running yet”.

All participants reported that reduced mobility impacted day-to-day function. Most patients with classic MPS VI were very limited in their ability to ambulate longer distances (greater than 1–2 blocks). None of the patients were able to participate in regular physical education at school. On outings, families had a range of strategies to deal with decreased ambulatory capacity including reducing the distance walked, carrying their child and using strollers, wheelchairs or scooters if available. Transitions from the floor to standing or standing to the floor were challenging for all patients with classic MPS VI.

In the cognitive debriefing session, participants found PODCI mobility items easy to understand and to provide a good impression of function. The multiple items assessing ambulation of varying distance, and items assessing walking up stairs, getting on and off the bus, getting off the toilet, and getting onto the bed were considered relevant and endorsed by participants. Caregivers specifically appreciated inclusion of items focused on sports, recreational activities, and physical education in school and the specificity of items identifying factors limiting participation in these activities.

On the PODCI Transfer and Basic Mobility, Sports and Physical Functioning, and the Global Functioning scales, most patients with MPS VI had normative scores at least 2 standard deviations (SD) below the normative mean. Exceptions were the youngest 4-year-old patient who had scores within 1 SD of the normative mean, and a 16-year-old patient with slowly progressing MPS VI who had a Transfer and Basic Mobility score within normal range and a Sports and Physical Function score at least 2 SD below the normative mean. This finding highlights the heterogeneous disease presentation in MPS VI and that in patients with slowly progressing MPS VI, mobility limitations have greater impact on more challenging activities, such as running and community sports and recreation participation although patients may still experience pain in their day-to-day mobility.

Seven of the 9 caregivers endorsed PROMIS Pediatric/Parent Proxy Bank v2.0—Mobility items involving walking up stairs, getting up from the floor, bending over to pick something up, getting into bed, and standing up from the toilet. Three additional items felt to capture meaningful functional impacts were “My child has been physically able to do the activities he/ she enjoys most”, “My child can do sports and exercise that other kids his/her age can do”, and “My child can keep up when he/ she plays with other kids”. Other mobility items, such as running a mile, getting in and out of a car, standing on tiptoes, were considered to be not relevant.

### Upper extremity and fine motor deficits

**Quote Fine Motor Dexterity and Managing Fasteners:** “We avoid jeans, we avoid zippers. Their winter coat is a poncho, they don't have to put their arms in it, it just goes over their head, and then they get in their car seat under the poncho. And, lots of leggings, slip-on shoes, crocs. T-shirts with lots of stretch because they can't…you can't do button-up, you can't do…if there's not stretch, there's no way”.

**Quote Fine Motor Dexterity and Managing Fasteners:** “As they're getting older, they don't want their mom or dad help them unzip and unbutton their pants for them. I have to help them button their pants at 18 years old, and they don't really like that. But, when they were younger, we did the elastic thing but now that they're older, they don't want to do that anymore.”

All participants (caregivers and patients) reported/self-reported limited arm and hand function. All patients with MPS VI had experienced carpal tunnel syndrome and several had undergone surgical repair. Managing clothing fasteners, such as buttons and zippers, was reported to be challenging. Putting on shoes and tying laces was difficult and was even more challenging if orthotics were worn. Decreased fine motor dexterity also impacted opening jars, which in addition to reaching overhead, was considered to be the most challenging upper extremity/fine motor activity by 4 caregivers and 2 patients (Table 3). Although writing was not considered the “most challenging” upper extremity/fine motor activity (Table 3), caregivers described writing as challenging. They noted that their children self-limited writing when possible, took breaks, and switched hands during writing. They also noted writing quality deteriorating over time.

**Table 3:** Upper extremity and fine motor activity considered to be the most challenging

	Caregiver	Patient
Pouring a drink from a full pitcher or carton	0	2 (28.5%)
Opening a jar by him or herself	4 (50%)	2 (28.5%)
Lifting or reaching overhead for a heavy item	4 (50%)	3 (42.9%)
Using a key to open a lock	0	0
Write with a pen or pencil	0	0
Total	8	7

Not all participants responded to polling questions because of fatigue, care provision, or other reasons

Patients with MPS VI completed the NIH Toolbox Pegboard Dexterity Test and found it to be feasible and relevant. Although challenging, this test was completed by all patients and was considered a relevant assessment of their fine motor deficits.

**Quote Decreased Shoulder ROM:** “I have, like, limited motion, so I can't lift things over my head. So, I have problems. I would say the biggest thing is, like, doing my hair. Like, it sounds like a simple task but I would say that's the biggest thing.”

Decreased shoulder range of motion (ROM) in patients with MPS VI was reported by 8 of the 9 caregivers and generally presented in the preschool years. Decreased shoulder ROM can have a significant impact on independence and ADL, potentially impacting dressing, bathing, and overhead reach. Four caregivers reported reaching overhead as the most challenging upper extremity/fine motor activity for their child (Table 3). Most participants reported that taking off a shirt was more difficult than putting it because of decreased shoulder ROM. Caregivers reported that their children developed compensatory strategies to get arms higher, such as overextending the spine.

In the cognitive debriefing session, 7 of the 8 items on the PROMIS Pediatric/Parent Proxy Short Form v2.0—Upper Extremity 8a were endorsed as being relevant, highlighting the daily challenges these patients face because of decreased hand and arm strength and dexterity. The single item that was not endorsed was the ability to use a key to unlock a door.

### Fatigue

**Quote Fatigue:** “I think it's relevant because fatigue is a symptom of MPS VI. I mean, it's a part of it, so I think that's a relevant thing…. It doesn't have to mean anything else, it just means you get wore out going to the mailbox, for no reason, you're just wore out.”

All participants (patients and caregivers) noted that fatigue, an overwhelming sense of tiredness, lack of energy, and feeling of exhaustion [12], was present and that it impacted ADL. Many of the patients took rests/naps or had to stop or modify activities because of an inability to keep up or complete tasks. The caregivers of the 2 younger children (aged 4 years) noted that their children may continue to participate in activities but would experience significant fatigue or pain following the activity. The older children had developed strategies to self-limit participation in activities. Key fatigue functional impacts were shortness of breath with ambulation and worsening fatigue with weather changes.

### Conceptual model and map to clinical outcome assessments

**Fig. 1:**
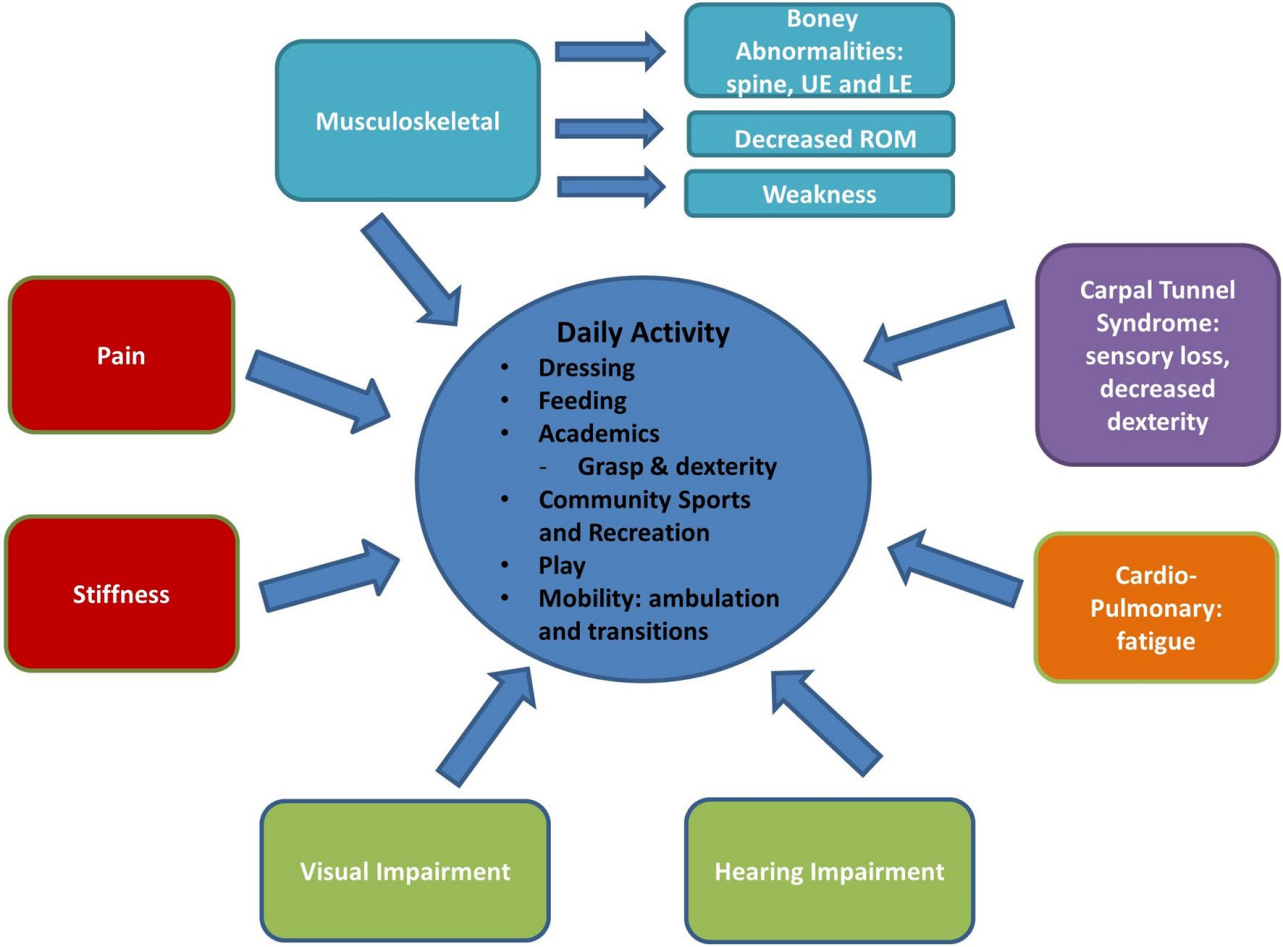
Conceptual model of key mucopolysaccharidosis type VI disease concepts and daily activity impacts. ADL: activities of daily living; LE: lower extremity; ROM: range of motion; UE: upper extremity

A conceptual model of key MPS VI disease concepts and functional impacts was developed (Fig. 1). Mobility limitations included transitions from floor to standing, stairs, getting up from the toilet, getting into bed, and walking distances longer than 1 to 2 blocks. Upper extremity functional challenges included dressing, overhead reach, lifting heavier items, and tasks that involve use of pincer grasp or whole hand grasp. Pain can affect mobility, ROM, upper extremity function, dressing, academics, peer interaction, and community sports and recreation. Fatigue can impact engagement in ADL in the home, community, and educational environment.

Functional impacts varied; patients with classic MPS VI had greater severity of boney abnormalities, more restricted ROM, and more stiffness than patients with slowly progressing MPS VI.

The MPS VI disease concepts of interest identified by the focus group were mapped to their functional impacts then to potential COAs considered appropriate for evaluating these impacts (Table 4). Key COA considerations included appropriateness to measure the disease concepts of interest (content validity), availability of normative data, which allows objective comparison of study population with age-matched, typically developing peers, previous use in disease-specific studies, and linking to key disease impairments (Table 5). In diseases with multisystem impairment and heterogeneity, using a range of COAs is often necessary to assess a broad range of functional impacts.

**Table 4:** MPS VI disease concepts of interest, functional impacts, and clinical outcome assessment

Disease concept of interest	Functional impact	Clinical outcome assessments
Impaired mobility	Difficulty managing mobility required for home and school environments Decreased ability to ascend/descend stairs, especially on school bus Inability to walk long distances; requires adapted equipment, such as strollers or wheelchairs Unable to participate in gym class at school Unable to keep up with peers in playground and in sports and recreation activities	Performance measures: 2MWT/6MWT Timed Stair Climb BOT™-2 Balance, Strength and Running Speed and Agility Subtests Patient Reported Outcomes: PROMIS® Mobility PODCI Transfer and Basic Mobility, Sports and Physical Function CHAQ PedsQL™
Upper extremity and fine motor deficits	Fine motor deficits impact dressing often resulting in need for adult assistance Handwriting difficulties Inability to reach overhead causes difficulty in ADLs, such as hair brushing, bathing and dressing Difficulty with tasks requiring both strength and dexterity such as opening a jar and managing clothing fasteners	Performance measures: NIH Toolbox Pegboard Dexterity Test Grip Strength BOT™-2 Fine Motor and Manual Dexterity Subtests Clinician Reported Outcome: Passive Range of Motion Patient Reported Outcomes: PROMIS® UE PODCI Upper Extremity CHAQ PedsQL™
Pain	Results in decreased HRQoL Limits ability to complete schoolwork Impacts sleep Limits ability to participate in sports and recreation activities	Patient Reported Outcomes: PROMIS® Pain Intensity, Pain Behavior, Pain Interference PODCI Pain and Comfort, Happiness CHAQ PedsQL™ Faces Pain Scale-Revised VAS NRS
Fatigue	Shortness of breath with ambulation Need for frequent rests and naps Unable to participate in typical age-appropriate activities	Performance measures: 2MWT/6MWT Patient Reported Outcomes: PROMIS® Fatigue PODCI Happiness, Pain and Comfort PedsQL™

*2MWT* 2-min walk test, *6MWT* 6-min walk test, *BOT-2* Bruininks–Oseretsky Test of Motor Proficiency, 2nd edition, *CHAQ* childhood health assessment questionnaire, *NIH* National Institutes of Health, *NRS* numeric rating scale, *PedsQL* Pediatric Quality of Life Inventory, *PODCI* Pediatrics Outcomes Data Collection Instrument, *PROMIS* Patient-Reported Outcomes Measurement Information System, *UE* upper extremity, *VAS* visual analog scale

**Table 5:** Clinical outcome assessments and considerations for their use in mucopolysaccharidosis clinical studies

COA	Age range	Disease concepts of interest	Additional considerations
2MWT [21]/6MWT [22]	≥ 3 years	Mobility Endurance Strength Fatigue	Self-paced walking test measuring distance walked in 6 min (6MWT) or 2 min (2MWT). An assessment of functional capacity in pulmonary, cardiac, and musculoskeletal systems Age specific normative data available [23–25] Multibody system assessment; difficult to assess which body system is responsible for change Previous use in MPS studies [18, 26]
3 Minute Stair Climb (3MSC)	Not defined	Mobility Endurance Strength Fatigue	Measures number of stairs climbed in 3 min. Assesses ambulatory capacity, strength, and endurance. More challenging motor task than ambulation on a flat surface Normative data not available (alternative is Timed Up and Down Stair Test for which normative data is available [27]) Variability may be present in the size and number of flights of stairs available at clinical sites Ceiling effect present if children are able to climb maximum number of available stairs Multibody system assessment; difficult to assess which body system is responsible for change Difficult to define meaningful change in the absence of normative data, especially in growing child Previous use in MPS studies [26, 28–30]
BOT-2 [31]	4 to 21 years	Strength Mobility UE Function Dexterity	Standardized assessment designed to provide an overview of fine and gross motor skills in people aged 4 to 21 years Generates norm-referenced scores across individual fine and gross motor subtests and composite scores. Can administer individual subtests that align with MPS VI disease concepts of interest Assesses higher gross motor function than walk test, with items to measure bilateral extremity coordination, balance, jumping, hopping and running Assesses numerous fine motor dexterity and precision items, such as cutting with scissors, copying shapes, and placing pegs in a pegboard Contractures can limit positioning for items such as pushups or sit-ups Age and functional level should be considered to determine if use is appropriate because floor effect may be present for lower functioning patients Limited use in MPS studies [32]
NIH Toolbox Pegboard Dexterity Test [21]	≥ 3 years	Dexterity UE Function	Measures the amount of time required to quickly place 9 small pegs into a pegboard and once completed, remove them NIH Toolbox provides normative reference data for children ≥ 3 years based on large diverse normative sample Captures the speed and accuracy of hand movements with manipulation of objects, which has functional relevance to daily activity in MPS VI Standardized administration, data collection and training materials available on NIH Toolbox app Use in MPS studies (ClinicalTrials.gov: NCT03370653)
NIH Toolbox Grip Strength Test [21]	≥ 3 years	Strength	Measures hand grip strength using a grip strength dynamometer NIH Toolbox provides normative reference data for children ≥ 3 years based on large diverse normative sample Standardized administration, data collection and training materials available on NIH Toolbox app Dynamometer must adjust for use with small hands Use in MPS studies [33, 34]
Passive Range of Motion	Any age	Limited joint flexibility	Normal references values available [35] Inter-rater reliability can be a challenge; this can be mitigated with training and standardized equipment [36] Stiffness can vary throughout day Previous use in MPS studies [6, 18, 37]
PROMIS® [11]	≥ 5 years Parent Proxy ≥ 5 years Self-report ≥ 8 years	Pain Mobility UE Function Dexterity Fatigue	Patient or parent proxy reported outcomes covers wide range of domains applicable to MPS VI including pain intensity, interference and behavior, fatigue, mobility, and UE function Normative reference values generated from general population and clinical disease samples Available in many languages Pain interference and pain behavior are observable so can be reported by proxy (caregiver) for children < 8 years of age Pain intensity is a self-report NRS and cannot be assessed in children < 8 years of age Previous use in MPS studies [38]
PODCI [10]	2 to 18 years Parent Proxy ≥ 2 years Self-report ≥ 11 years	Mobility Dexterity Pain Fatigue HRQoL	Patient or parent proxy reported outcome designed to assess change following pediatric orthopedic interventions in a wide range of diagnoses [39] Standardized scores calculated from 0 to 100 with higher scores representing less disability and better function. Standard scores were also normed on US population to create normative scores for each scale PODCI constructs applicable to MPS VI include UE function, transfers and mobility, physical function and sports, and comfort (lack of pain) The Physical Function and Sports domain provides unique and MPS VI relevant content related to community, sports, and recreation participation Versions available in Korean, Spanish and English [40] Previous use in MPS studies [41]
CHAQ [42]	Ages for Parent Proxy and Self-report not specified	Pain Mobility UE Function Dexterity Fatigue	Patient or parent proxy reported outcome designed to measure health status and physical function in juvenile arthritis. Has been validated for use in children with chronic musculoskeletal pain [40] Normative data is not available. A Disability Index (DI) is calculated by pooling domain scores, with higher scores reflecting greater disability. Pooling of domains may limit ability to interpret primary disease concept of change Items that cannot be completed because they are not developmentally appropriate are left blank resulting in fewer items to detect change in younger patients CHAQ constructs relevant to MPS VI are physical function, dressing and grooming, eating, walking, hygiene, reach and grip Includes Visual Analog Pain Scale Original CHAQ translated and validated in over 40 languages [40] Previous use in MPS studies [6, 18, 43]
PedsQL™ [44, 45]	≥ 2 years Parent Proxy ≥ 2 years Self-report ≥ 5 years	Pain Mobility UE Function Fatigue HRQoL	Patient or parent proxy reported outcome designed to measure HRQoL in children and adolescents with acute and chronic health conditions. Numerous modules are available to capture constructs relevant to MPS VI including the Generic Core Scales, the Family Impact Module, and the Multi-Dimensional Fatigue Scale Scored on a scale of 1 to 100 where higher scale scores indicate better HRQoL and distinguishes healthy children from children with health conditions but does not provide comparison to age specific normative data Covers many domains with few questions for each, resulting in less information available for each domain Available in many languages Previous use in MPS studies [8, 46]
Faces Pain Scale-Revised [47]	≥ 4 years	Pain	Self- report of pain intensity developed for children. Children choose the face that best illustrates the pain they are experiencing Allows self-report of pain for young children Easy to administer Available in many languages Previous use in MPS studies [8]
VAS for Pain	≥ 7 years	Pain	Horizontal line 10 cm in length. On one end the descriptor is ‘no pain’ and on the other end the descriptor is ‘very severe pain’. The subject marks a spot on the line that represents their pain level within a given recall period CHAQ includes a VAS Appropriate for ages 7 and older [48] Previous use in MPS studies [8, 42]
NRS for Pain	≥ 8 years	Pain	Numeric scale from 0 to 10 on which patients estimate their pain numerically, with higher numbers representing increasing pain severity PROMIS Pediatric Numeric Rating Scale v1.0-Pain Intensity is a NRS [11] Appropriate for ages 8 and older [48] Limited use in children with MPS [49]

*2MWT* 2-min walk test, *6MWT* 6-min walk test, *ADL* activities of daily living, *BOT-2* Bruininks–Oseretsky Test of Motor Proficiency, 2nd edition, *CHAQ* childhood health assessment questionnaire, *COA* clinical outcome assessment, *DI* disability index, *HRQoL* health-related quality of life, *MPS* mucopolysaccharidosis, *NIH* National Institutes of Health, *NRS* numeric rating scale, *PedsQL* pediatric quality of life inventory, *PODCI* pediatrics outcomes data collection instrument, *PROMIS* patient-reported outcomes measurement information system, *UE* upper extremity, *VAS* visual analog scale

## Discussion

Participants in the MPS VI focus group identified pain, impaired mobility, upper extremity and fine motor deficits, and fatigue as the most important disease concepts of interest experienced by pediatric patients with MPS VI. The impacts of these concepts permeated the patients’ daily lives, affecting their independence and capacity to participate in age-appropriate activities. These concepts were prominent despite all patients who participated in the focus group receiving ERT (up to 13 years in some patients).

Pain is common in patients with MPS, a sentiment confirmed by both caregivers and pediatric patients in our focus group. Pain was reported in hands, wrists, shoulders, knees, hips and back; impacted sleep, participation in sports/recreation, and completion of schoolwork; and resulted in functional deficits and lower HRQoL. Pain and function are inextricably linked; increased pain in pediatric rheumatic disorders is associated with increased functional impairment [13]. Not surprisingly, HRQoL is also impacted by pain. In a study of pain in patients with MPS [8], higher pain scores correlated with lower scores on the Pediatric Quality of Life Inventory (PedsQL™) psychosocial scale.

Knowledge of the etiology of pain in MPS, particularly in pediatric patients, is sparse [14]. Localized tissue injury or inflammation (ie, nociceptive pain) resulting from accumulation of glycosaminoglycan in soft tissue, organs, and joint spaces has been implicated, as has neuropathic pain caused by carpal tunnel syndrome and MPS-related musculoskeletal injury/abnormalities, such as cervical spinal stenosis [14]. Despite being common, pain is underestimated in MPS patients, and standardized pain assessments are recommended as part of standard care [8].

Pain can be measured in many ways. Pain intensity is preferably reported by the patient because it is not observable by a proxy. Patient-reported pain intensity using visual analog scales is typically possible from 7 years of age [15], numerical rating scales by 8 years of age [16], and assessments such as the Faces Rated Pain Scale-Revised can be used from 4 years of age [17]. In contrast, pain interference, which characterizes the impact of pain on function, and pain behavior which describes ways that patients cope with and respond to pain are both observable pain behaviors and can be reported by a proxy and the patient.

Joint pain, stiffness, and inflammation contribute to upper extremity and fine motor deficits and manifest in reduced independence in ADL, such as dressing, bathing, and food preparation. The upper extremity and fine motor deficits reported by this focus group are consistent with those reported in a study by Swiedler et al. [18]. In this study, patients with MPS VI and ≤ 18 years of age (n = 91) had a Childhood Health Assessment Questionnaire (CHAQ) Disability Index score indicating a moderate level of disability. This score signified patients had difficulty with dressing, hygiene, grip, and daily activities, as well as arising, eating, and walking.

Patient-reported outcomes (PROs), such as the CHAQ, PedsQL, PODCI, and PROMIS Upper Extremity can be used to ask patients about their ability to complete tasks, such as dressing, bathing, opening a jar, writing with a pencil, and reaching overhead. In addition to PROs that measure decreased joint flexibility and decreased dexterity, the NIH Toolbox Pegboard Dexterity Test and joint ROM measurements are additional ways to measure these concepts of interest. Reliable joint ROM measurement can be challenging because of a patient’s degree of stiffness may vary throughout the day and interrater reliability. These issues may be mitigated by standardized assessor training, uniform equipment used at all sites, and ensuring that ROM is measured at the same time of day at each assessment.

The focus group reported impaired mobility, which impacted ambulation over long distances, regular participation in physical education at school, and multiple aspects of daily function in the home, school, and community. The inability to keep up with one’s peers is detrimental to peer relationships and social connectedness. Reduced long-distance mobility leads to activity modifications, reduced participation, and increased need for adaptive equipment. In a study of adult and pediatric patients with a broad clinical spectrum of MPS VI, 23% (27/120) required a device as an aid for mobility (eg, wheelchair, walker, cane, crutches) [18]. Although devices, such as wheelchairs, can mitigate the impact of impaired mobility, the cost, availability, and acceptance of these devices by the patient, family and peers can be barriers for their use.

Mobility skills can be measured in pediatric patients directly, using the 2- or 6-min walk tests and timed stair climbs, and in PROs, using CHAQ, PROMIS Mobility, PODCI, and PedsQL. These PROs ask patients questions regarding their mobility skills, such as their level of difficulty in walking outdoors on flat ground, walking up and down stairs, and bending down to pick up something from the floor. The PODCI is one of the few PROs that assess participation in sports and recreation activities.

Fatigue is a frequently cited symptom in MPS; however, research assessing fatigue in MPS VI is minimal [4]. Fatigue was reported as an issue by all focus group participants and impacted daily life. Many of the patients took rests or naps, or discontinued or modified activities due to an inability to keep up or complete the tasks. Whereas older children often developed strategies to pace themselves and self-limit participation, younger children reportedly had not yet developed this strategy, and overexertion resulted in increased pain and fatigue. Gold et al. [19] found fatigue to be closely associated with chronic pain and to act as a mediator between pain and overall HRQoL, suggesting fatigue may be a critical symptom that helps explain the relationship between chronic pain and quality of life. Fatigue can be measured directly by the patient’s ability to complete physical activities, such as walk tests, and can be captured in PROs, such as PedsQL, PROMIS Fatigue, and PODCI.

The cumulative and combined impact of decreased upper extremity strength, flexibility and dexterity affect the ability of children with MPS VI to function independently across many domains. Parental assistance is typical and well accepted in younger children but as children age, their independence generally increases and social role changes. The desired independence of an adolescent contrasts with the level of assistance required from caregivers due to MPS VI disease progression.

Engaging patients and caregivers with rare diseases for their input on how their disease impacts their day-to-day life is critical. A potential limitation of this project may be the relatively small sample size. Identifying the most burdensome functional impacts allows consideration of the most appropriate COAs to measure the impacts. Selection of COAs for clinical trials in pediatric rare diseases has unique challenges, such as wide age range and heterogeneous disease presentation. In addition, the age-related variability of motor skills and daily activity make it difficult to distinguish between treatment effect and developmental maturity [20]. Existing standardized developmental assessments can provide normative values to characterize disease presentation and to measure treatment effect. Considerations for and examples of potentially appropriate COAs for MPS VI are highlighted (Table 5); however, an in-depth analysis of each COA’s properties was not within scope.

## Conclusion

In this project, caregivers and patients with MPS VI provided valuable input on disease symptoms and the impact on daily life to inform COA selection and endpoint design for clinical trials in MPS VI. Overall, caregivers and patients endorsed that although the children receive standard of care ERT, residual symptoms exist and impact HRQoL. The key concepts of interest highlighted were impaired mobility, upper extremity and fine motor deficits, pain, and fatigue. Pain was unanimously reported by all patients, across many areas of the body and impacted daily activity, sleep, school function, and community participation.

Key MPS VI disease concepts were mapped to a selection of pediatric COAs with a specific focus on generic measures, such as the PROMIS, PODCI, CHAQ and the PedsQL. Caregivers endorsed the relevance of the PODCI and PROMIS Upper Extremity, Mobility, and Pain items and all children completed the NIH Toolbox Pegboard Dexterity Test. Additional COAs that align with the MPS VI disease concepts include range of motion, the 2- and 6-min walk tests, timed stair climbs, Bruininks-Oseretsky Test of Motor Proficiency, 2nd edition, grip strength, pain visual analog scale and the faces pain scale-revised.

## Data Availability

Data archiving is not mandated but data will be made available on reasonable request.

## Abbreviations

ADL: Activities of daily living
CHAQ: Childhood Health Assessment Questionnaire
COA: Clinical outcome assessment
ERT: Enzyme replacement therapy
HRQoL: Health-related quality of life
MPS VI: Mucopolysaccharidosis type VI
NIH: National Institutes of Health
PedsQL: Pediatric Quality of Life Inventory
PODCI: Pediatric Outcomes Data Collection Instrument
PRO: Patient-reported outcome
PROMIS: Patient Reported Outcome Measurement Information System
ROM: Range of motion
SD: Standard deviation
